# Impact of the biological definition of Alzheimer’s disease using amyloid, tau and neurodegeneration (ATN): what about the role of vascular changes, inflammation, Lewy body pathology?

**DOI:** 10.1186/s40035-018-0117-9

**Published:** 2018-05-31

**Authors:** S. Gauthier, H. Zhang, K. P. Ng, T.A. Pascoal, P. Rosa-Neto

**Affiliations:** 1 McGill Center for Studies in Aging, Douglas Mental Health Research Institute, Montreal, Canada; 2 Department of Neurology, The First Affiliated Hospital of Chongqing Medical University, Chongqing, China; 3 Department of Neurology, National Neuroscience Institute, Singapore, Singapore

**Keywords:** Alzheimer’s disease, Diagnosis, Treatment, Biomarkers, Precision medicine, Translational research, Brain imaging, Database analysis, Human volunteer cohorts

## Abstract

**Background:**

The NIA-AA research framework proposes a biological definition of Alzheimer’s disease, where asymptomatic persons with amyloid deposition would be considered as having this disease prior to symptoms.

**Discussion:**

Notwithstanding the fact that amyloid deposition in isolation is not associated with dementia, even the combined association of amyloid and tau pathology does not inevitably need to dementia over age 65. Other pathological factors may play a leading or an accelerating role in age-associated cognitive decline, including vascular small vessel disease, neuroinflammation and Lewy Body pathology.

**Conclusion:**

Research should aim at understanding the interaction between all these factors, rather than focusing on them individually. Hopefully this will lead to a personalized approach to the prevention of brain aging, based on individual biological, genetic and cognitive profiles.

## Background

The treatment of Alzheimer’s disease (AD) is currently symptomatic and based on neurotransmitter manipulation, akin to what has been achieved in Parkinson’s disease. Thus acetycholine activity is being increased by cholinesterase inhibitors, and glutamatergic activity is being dampened by memantine action on NMDA receptors. A modest but clinically detectable response is present in many patients using such drugs alone or in combination.

Unfortunately the next generation of drugs acting on AD core pathological factors such as amyloid deposition and phosphorylated tau aggregation has failed so far to delay disease progression, raising the issue of timing of these interventions along the continuum of AD neurodegeneration over time.

This review wants to highlight the facts that other pathological factors are at play in AD, and deserve consideration in the full diagnostic assessment of the patients, and for treatment. These factors are vascular changes, Lewy body pathology and neuroinflammation.

### Classic pathology of AD

The clinical progression of AD is linked to specific neuropathological features, such as extracellular deposition of Aβ plaques, intracellular inclusions of tau protein in neurofibrillary tangles, and neuronal degeneration. The discovery and advance of disease biomarkers over the last decade have significantly advanced our understanding of the dynamic pathophysiological changes underlying AD and have allowed the detection of AD pathophysiology in vivo [1]. Given that the presence of AD pathophysiology has been found across a broad clinical spectrum including individuals asymptomatic and with mild cognitive symptoms, biomarkers now play an important role in characterizing the trajectory of AD pathophysiology and have been incorporated in the AD diagnostic research criteria [2–5]. These diagnostic research criterions recognize that the coexistence of abnormal Aβ and tau biomarkers better identify the preclinical and MCI individuals who will progress to dementia over relatively short time frames of three to 5 years.

Based on histopathological and genetic evidences, fibrillar Aβ, the main constituent of Aβ plaques, has been postulated as the major driving force leading to AD dementia (Aβ cascade hypothesis). According to this hypothesis, all the resulting pathological processes are due to an imbalance between Aβ production and clearance, which would then potentiate the spread of tauopathy, leading to neurodegeneration and cognitive decline. However, the lack of consistent association between Aβ and clinical progression, and the fact that amyloid pathology has been found in cognitively normal elderly individuals challenge the Aβ hypothesis in its original form.

### Proposal for a new classification system

An unbiased biomarker classification system, A/T/N, which avoids the assumptions of the temporal ordering of AD biomarkers, has been proposed [6]. In this classification system where each biomarker category is binarized as either positive or negative, “A” represents Aβ biomarkers using amyloid PET or CSF Aβ_42_, “T” represents tau biomarkers using CSF p-tau or tau PET, and “N” represents neurodegeneration biomarkers using CSF p-tau, structural MRI or [^18^F]fluorodeoxyglucose PET (FDG). This descriptive classification aims to organize the multi-modality biomarker results at the individual person level in a way that is easy to adopt and interpret. Other brain pathological processes have been postulated as natural candidates to integrate this unbiased system. Studies under way are measuring simultaneously the amyloid, tau, and neuroinflammation in individuals, with follow-up over time to test the hypothesis that the coexistence of the brain pathological factors may accelerates AD clinical manifestations.

We argue that the A/T/N classification may be broadened to include other key pathological factors: vascular pathology, Lewy Body pathology and neuroinflammation.

### Vascular changes

There is growing evidence that AD often coexists with cerebrovascular disease (CVD). They share many risk factors, leading to additive or synergistic effects on cognitive decline [7]. The APOE ε4 allele is the strongest genetic risk factor for late-onset AD, and APOE ε4 is also associated with increasing burden in MRI markers for both ischemic and hemorrhagic CVD [8]. There is increasingly robust relationship between other risk factors including hypertension, diabetes, atrial fibrillation, hypercholesterolemia, smoking, hyperhomocysteinaemia, age and obesity and AD, whereas there are possible protective effect of the ‘Mediterranean’ diet and physical exercise [9–14]. Although not all studies have found a correlation between vascular risk factors and AA [15, 16], it has been reported that the presence of vascular risk factors can predict the development of AD or the conversion from mild cognitive impairment (MCI) to AD [9, 17, 18].

Most AD patients have structural changes in their cerebral blood vessels. Imaging and pathological studies have demonstrated a high prevalence of arteriolosclerotic small vessel disease (SVD) in AD patients. Post-mortem and imaging studies demonstrate that arteriolar Aβ amyloid angiopathy, a sub-type of SVD, is more common in patients with AD than in elderly controls [19–23]. The amyloid angiopathy mainly affects the leptomeningeal, cortical and capillary vessel walls, but sometimes the cerebellum, and occasionally the brainstem [12, 24]. In the autopsy studies, it suggests that AD is correlated with atherosclerosis of the Circle of Willis, and the severity of the atherosclerosis is associated with neuritic plaques and neurofibrillary tangles [25–27].

An important component of CVD in AD is cerebral hypoperfusion, which can be present several years before the onset of clinical symptoms. The diffusion pattern of cerebral hypoperfusion is stereotyped in AD: the first affected area of is the precuneus, which has appeared 10 years before the onset of AD, followed by the cingulate gyrus and the lateral part of the parietal lobe, then the frontal and temporal lobes, and the eventually the cerebrum [12]. The main mechanism of cerebral hypoperfusion in AD may be non-structural [12]. In vivo and in vitro studies have shown cerebral hypoperfusion increases the production of Aβ and tau hyperphosphorylation, reduces the clearance of Aβ, then aggravates the progress of AD [28–33]. There is good evidence that Aβ amyloid angiopathy and SVD are associated with infarction and cerebral hemorrhage in AD [34–43]. The mechanisms may involve susceptibility to thrombosis, reduction of blood flow, impaired caliber regulation, and impaired function of the blood-brain barrier (BBB). Infarction or bleeding will reduce the threshold for the onset of AD, and is considered as an important risk factor for the clinical manifestations of AD [44, 45].

The links between vascular factors and AD have been clearly confirmed both clinically and pathologically. However, there is a lack of high-quality therapeutic research to examine the extent to which vascular risk changes alter the course of AD. Further longitudinal mechanisms and therapeutic studies are needed, especially to determine whether the treatment of vascular risk factors can prevent or delay the onset of AD.

### Lewy body pathology

Although the accumulation of amyloid protein in plaques and tau protein in neurofibrillary tangles constitutes the core pathological feature of AD, the presence of abnormal brain aggregates of a third proteinopathy has been shown to be very prevalent in moderate and severe AD [46–48]. Cytoplasmic inclusions of α-synuclein intraneuronally in Lewy bodies have been reported in up to 50% of sporadic AD cases and up to 60% of familial AD cases [49–52]. In the context of AD, it is still unclear whether the overlap between Lewy bodies and the hallmark AD proteins occurs due to a mere co-occurrence of independent pathological processes or is the manifestation of interconnected pathological processes.

Histopathological studies have shown that Lewy bodies normally accumulate in a specific topographic brain pattern, starting in the brainstem and subsequently extending to the limbic and neocortical brain regions [47]. In contrast, in AD patients the Lewy bodies deposition concentrates in the amygdala with little deposition in the brainstem or neocortex [53]. This characteristic pattern of deposition has been called AD with amygdala Lewy bodies [47, 54]. Interestingly, the Lewy bodies in the amygdala normally overlap with tau accumulation [55] and neuronal loss [56], suggesting that pathological interaction between these pathologies may play a role in the progression of AD. The severity of the pathology in the amygdala correlates with disease duration [55, 56] and emotional and memory difficulties [57], which suggest that the aforementioned interaction plays a role in the AD clinical phenotype in this group of individuals [58]. Moreover, the cortical concentrations of Lewy bodies have been correlated with amyloid burden [59, 60] and neurofibrillary tangles [61, 62].

Postmortem observations focusing on the influence of Lewy bodies and the phonotypical presentation of AD have shown inconsistent results. These studies presented opposing results whether the presence of Lewy bodies in AD patients has an effect on the age of onset of symptoms, death [51, 63–65], the likelihood of be an APOE ε4 carrier [51, 65, 66], parkinsonian symptoms [63, 64, 67, 68], cognitive impairment [63, 64], or visual hallucinations [64, 68–70]. This disagreement arises in part, due to the fact that most of these studies have small sample sizes or limited range of AD phonotypical presentations. However, it is worth to mention that a well-powered multicenter study with a high sample size has reported that the onset of symptoms and death in AD individuals with Lewy bodies occurs at younger ages as compared to those without Lewy bodies, and that AD individuals with Lewy bodies have higher chance to be APOE ε4 carriers than AD individuals without Lewy bodies. Moreover, this study also suggested that hallucinations, motor disturbs, and sleep problems are more severe in AD individuals with Lewy bodies than in the ones without Lewy bodies [51].

The α-synuclein protein, which in the main constituent of the Lewy bodies, can be measured in the cerebrospinal fluid (CSF) of living people [71]. Some CSF studies have reported an increase in α-synuclein levels in patients with MCI and AD as compared to controls [72–74], whereas other studies have shown no difference or reduced levels across the AD clinical spectrum [75–77]. The levels of α-synuclein have shown positive correlation with CSF tau pathology in some studies [73, 77] and no correlation in another [78].

Although the characteristic topographic presentation and the frequency of Lewy bodies in AD suggest a potential common mechanism for AD and Lewy bodies, the divergence in results between studies indicates that further studies are imperative to clarify the role of Lewy body pathology in AD. One of the main limitations of the current studies is the absence of an imaging agent able to capture Lewy bodies in the living brain. Many groups are trying in developing such an imaging agent in order to provide the means to definitively clarify the dynamical changes of Lewy bodies in the human brain and its interplay with amyloid, tau, neuroinflammation, and vascular pathology.

### Neuroinflammation

In addition to hallmark AD neuropathological features such as amyloid (Aβ) plaques, neurofibrillary tangles and neuronal degeneration, there is a growing body of evidence supporting neuroinflammation as an important player in the pathogenesis of AD [79, 80]. Neuropathological studies have shown the presence of activated microglia and inflammation related mediators in AD brains of low Braak stage [81], while genetic studies show that several genes that increase the risk of sporadic AD encode factors that regulate microglial clearance of misfolded proteins and inflammatory reaction, such as TREM2 and CD33 [82, 83]. Epidemiological studies further suggest that non-steroidal anti-inflammatory drugs (NSAIDS) can defer or prevent the onset of AD [84, 85]. Although subsequent clinical trials involving prednisolone and NSAIDS, such as the Alzheimer’s Disease Anti-inflammatory Prevention Trial (ADAPT), failed to show improvement in cognitive decline in AD patients or prevent AD progression in adults with a family history of dementia [86], the difference between observational and randomised studies will need to be clarified in future studies.

Microglia, the resident phagocytes of the brain, plays an integral role in maintaining brain homeostasis and protecting the brain from insults by mounting an innate immune response when activated [87]. Preclinical and post-mortem studies have consistently found that activated microglia colocalises with Aβ plaque [88, 89], suggesting a close intimate relationship between microglia activation, Aβ and neuroinflammation. In AD, microglia bind to soluble Aβ oligomers and fibrils via cell surface receptors, which triggers the activation of microglia [80]. A key issue is whether this response is adaptive or maladaptive in nature. While acute microglia activation triggered by Aβ is aimed to eliminate Aβ aggregation via phagocytosis, there is an inefficient clearance of Aβ plaques [90]. Several mechanisms have been hypothesised, including ongoing formation of Aβ and positive feedback loops between inflammation and amyloid precursor protein (APP) processing which compromise the cessation of neuroinflammation. Continued exposure to Aβ, chemokines, cytokines, and inflammatory mediators leads to microglia being chronically activated at the Aβ plaque site, which further contribute to Aβ production and accumulation in a vicious cycle.

Microglia and neuroinflammation are also closely associated with tau in AD. Reactive microglia can produce inflammatory cytokines such as IL-1 which lead to an increase in tau phosphorylation in neurons [91]. This may contribute to the development of tau pathology and thus accelerate the course of disease. Furthermore, misfolded tau may also trigger microglial activation [92]. In preclinical studies, reactive microglia are found to be sufficient in driving tau pathology and contribute to the spread of pathological tau in the brain [93]. Microglia have also been shown to internalize tau protein both in vitro and in vivo. In post-mortem studies, microglia colocalise with various forms of tau in brain tissue of AD patients [94].

Given the dynamic relationship between Aβ, tau and microglia in AD, it is imperative to study the interplay between these pathophysiologies so as to further understand the sequence of events underlying the AD process. In this regard, studies that measure Aβ, tau, and neuroinflammation concurrently will be of paramount importance. The findings of these studies will further broaden the A/T/N classification of individuals to include neuroinflammation biomarkers.

## Conclusion

Towards an integration of the various pathological factors leading to targeted treatments.

This expanded view of the pathological factors at play in persons with AD may be lead to therapeutic strategies targeting the most active factors at a given time in each individual. We hope that meta-analysis of current observational studies such as ADNI and others under development such as COMPASS-ND will facilitate the validation of various imaging, CSF and blood markers for each of these pathologies, as illustrated in Table 1. In other words “mixed dementia” which is most common finding in autopsy studies will be in the near future be studied based on biomarkers. This may allow for more homogeneous groups of patients to be studied in randomized clinical trials require combination therapy, as a first step towards a personalized approach to treatment of AD throughout its course.

**Table 1:** Study of the various known pathological factors in AD

Factor	Imaging	CSF	Blood	Potential RX
Amyloid-β load	[^11^C]PIB [^18^F]NAV4694 [^18^F]florbetapir [^18^F]florbetaben [^18^F]flutemetamol	Amyloid-β(1–42)	APP669–711; Amyloid-β(1–42); Amyloid-β(1–40);	BACE inhibitors Amyloid-β immunotherapy
Neurofibrillary tangles load	[^18^F]MK6240 [^18^F]AV1451 [^11^C]PBBB3	Phosphorylated tau	The association of serum phosphorylated tau with tangles is unclear	Anti-aggregation Tau immunotherapy
Neurodegeneration	MRI [^18^F]FDG	Neurofilament light chain (NFL); d neurogranin (Ng); Visinin-like protein-1 (VILIP-1); Synaptosomal-associated protein 25 (SNAP-25); Neuron-specific enolase (NSE); Heart fatty acid binding protein (HFABP)	Neurofilament light chain (NFL)	Neurotrophic factors
Vascular load	MRI	CSF albumin /plasma albumin ratio		Control of risk factors
Lewy Body load	NA	α-synuclein	α-synuclein	α-synuclein immunotherapy
Neuroinflammation activity	Microglial Activation: [^11^C]PK11195 [^11^C]PBR28 [^11^C]DAA1106 [^18^F] DPA714 [^11^C] DPA713 [^18^F]GE180 Reactive astrocytes: [^11^C]L-des-deprenyl	Microglial Activation: Chitinase-3-like protein 1 (YKL-40), soluble TREM2 (sTREM2) Cytokines: TNF-α, IL-6, IL-1β Chemokines: monocyte chemotactic protein 1 [MCP-1]	Microglial Activation: Chitinase-3-like protein 1 (YKL-40) Cytokines: TNF-α, IL-6, IL-1β, Chemokines: monocyte chemotactic protein 1 [MCP-1]	NSAIDS Peroxisome proliferator-activated receptor-γ (PPAR-γ) activators TNF-α inhibitor
